# Dynamic Function and Composition Shift in Circulating Innate Immune Cells in Hibernating Garden Dormice

**DOI:** 10.3389/fphys.2021.620614

**Published:** 2021-03-04

**Authors:** Nikolaus Huber, Sebastian Vetter, Gabrielle Stalder, Hanno Gerritsmann, Sylvain Giroud

**Affiliations:** ^1^Research Institute of Wildlife Ecology, Department of Interdisciplinary Life Sciences, University of Veterinary Medicine Vienna, Vienna, Austria; ^2^Unit of Veterinary Public Health and Epidemiology, Institute of Food Safety, Food Technology and Veterinary Public Health Department for Farm Animals and Veterinary Public Health, University of Veterinary Medicine Vienna, Vienna, Austria; ^3^Institute of Animal Welfare Science, Department for Farm Animals and Veterinary Public Health, University of Veterinary Medicine Vienna, Vienna, Austria

**Keywords:** torpor, metabolic depression, arousal, oxidative burst, immunity, hibernator, ROS

## Abstract

Hibernation is characterized by successive torpor bouts during which metabolic rate is down-regulated to 2–4% of euthermic levels along with core body temperatures (T_*b*_) ranging between 0 and 10°C. One characteristic of the torpid state, which is periodically interrupted by a few hours of euthermic phases or arousals during hibernation, resides in an overall impairment of the immune system. The most striking change during torpor is the reduction of circulating white blood cells up to 90%, while their numbers rise to near summer euthermic level upon rewarming. However, potential changes in responsiveness and function of neutrophil granulocytes, accounting for the primary cellular innate immune defense, are unknown. Here we present the first data on shifts in oxidative burst capacity, i.e., the ability to produce reactive oxygen species (ROS), of neutrophils during hibernation. Using a chemiluminescence assay, we measured real-time ROS production in whole blood of hibernating garden dormice (*Eliomys quercinus*) in early or late torpor, and upon arousals. Accounting for changes in neutrophil numbers along the torpor-arousal cycle, we found significant differences, between torpid and euthermic states, in the neutrophil oxidative burst capacity (NOC), with shallow cell responses during torpor and a highly significant increase by up to 30-fold during arousals. Further, we observed a significant reduction of NOC from aroused animals with euthermic T_*b*_ of 36.95 ± 0.37°C, when tested at 6°C, whereas no change occurred in NOC from torpid individuals reaching constant T_*b*_ of 4.67 ± 0.42°C, when measured at 35°C. This dynamic indicates that the reduction in NOC during torpor may be temperature-compensated. These results linked to the understanding of immune function during the torpor-arousal cycle might have clinical relevance in the context of therapeutic hypothermia and reperfusion injury.

## Introduction

Endothermic vertebrates sustain a high basal metabolism to maintain a stabilized body temperature (T_*b*_). Confronted with large fluctuations in ambient temperature (T_*a*_) and bottlenecks in food (energy) and water availabilities a wide array of physiological and behavioral adaptations to conserve energy have evolved (Terrien et al., 2011; Boyles et al., 2016). The prime example of these adaptive strategies is hibernation or multi-day torpor (Lane, 2012; Ruf and Geiser, 2015) which is described in a surprisingly wide range of species and is considered the most effective means of energy conservation in endotherms (Geiser, 2013; Boyles et al., 2020). Hibernation is characterized by successive bouts of torpor, lasting for days or even weeks, where the metabolic rate is actively down-regulated to 2–4% of euthermic rates with a subsequent drop of core T_*b*_ reaching values between 0 and 10°C during torpor. During the ultra-metabolic downstate of torpor, all essential physiological systems including the cardiovascular function, respiration, digestive system, brain and renal metabolism as well as cellular mitosis are profoundly reduced (Carey et al., 2003; Storey, 2010; Jastroch et al., 2016; Jørgensen et al., 2020). Remarkably, in most hibernators, hibernation is interspersed by short periods of less than a day, called interbout arousals, during which animals quickly (<90 min) increase their metabolism and return to euthermic T_*b*_ levels while restoring their main physiological functions (Carey et al., 2003; Heldmaier et al., 2004; Ruf and Geiser, 2015; Jastroch et al., 2016). Although the organism of hibernating mammals undergoes these extreme and rapid metabolic and temperature changes, no evidence of organ injury can be found (Dugbartey et al., 2018). Therefore it has been suggested that hibernating mammals represent a natural model to study tissue injury following surgery, trauma or transplantation in the context of ischemia and reperfusion injury. The primary pathomechanism of ischemia and reperfusion injury is the recruitment of innate immune cells such as leukocytes and the formation of cytotoxic reactive oxygen species (ROS; Thiele et al., 2018).

The immune system is one of the vital physiological compartments that is severely affected by extreme shifts in physiological states during the torpor-arousal cycle (Bouma et al., 2010a; Bouma et al., 2011). Several studies reported an overall impaired function of the innate as well as the cellular and humoral adaptive immunity during torpor, including lower complement levels, reduced macrophage phagocytic capacity, diminished lymphocyte proliferation as well as decreased cytokine and antibody production (reviewed by Bouma et al., 2010a, 2012, 2013b). In this context, the most striking immunological change is the drop of circulating white blood cells (WBC) by up to 90%. In all investigated torpid or hibernating animals WBCs including neutrophil granulocytes, lymphocytes and monocytes are decreased, whereby neutrophil granulocytes, hereafter referred to ‘neutrophils’, remain the most abundant (Bouma et al., 2010a; Sahdo et al., 2013; Uzenbaeva et al., 2019).

In mammals, neutrophils are part of the first line of cellular innate immune defense against invading pathogens (Mantovani et al., 2011; Dahlgren et al., 2019). Activated neutrophils generate and release superoxide molecules, also called oxygen free radicals, as the basis for several anti-pathogenic and highly ROS which are also involved in other biomedical aspects such as the integration and resolution of inflammation and tissue injury and repair (Thiele et al., 2018; Yang et al., 2019; Dinauer, 2020). The induced ROS production is accompanied by a steep increase in cellular oxygen consumption, also referred to as “oxidative burst.” In particular, the use of whole blood chemiluminescence is a highly effective means for quantifying leukocyte (neutrophil) function, as reported by several studies measuring the production of ROS species (Lilius and Marnila, 1992; Kukovetz et al., 1997; Shelton-Rayner et al., 2012; Dahlgren et al., 2020).

During hibernation, the extreme up-shift in metabolic functions upon interbout arousal is paralleled by a vast increase in circulating WBCs, close to levels observed during summer euthermia (Bouma et al., 2011, 2013a). Contrary to shifts in WBC abundance and composition, there is, however, very little known on their responsiveness and functionality along the torpor-arousal cycle. Novoselova et al. (2000) showed in Arctic Yakutian ground squirrels (*Citellus undulatus Pallas*) that the production of tumor necrosis factor-alpha (TNFα) of macrophages, another circulating cellular component of the innate immune system, is significantly reduced during torpor, but restored to summer euthermic levels upon arousals. Further, the authors reported no change in the production of TNFα over the entire season in splenic T-lymphocytes (Novoselova et al., 2000). To the best of our knowledge, insight on the responsiveness and function in one of the most abundant circulating immune cells, i.e., neutrophils, during hibernation is lacking.

Therefore, in the present study, we aimed at (i) quantifying the number of different circulating WBC populations and (ii) determining the responsiveness of neutrophils within the torpor-arousal cycle as it occurs at mid-hibernation. Specifically, we hypothesized that, along with the marked reduction of neutrophils during torpor, the ability of neutrophils to produce oxygen free radicals would be dampened to a minimal level at low T_*b*_. We further hypothesized that the function of neutrophils would be recovered to a certain degree upon arousals. To test these hypotheses, we used a whole blood chemiluminescence assay to measure real-time neutrophil oxidative burst capacity (NOC) along the torpor-arousal cycle of the garden dormouse (*Eliomys quercinus*), a medium-sized hibernating rodent. To assess differences in circulating WBC composition and potential shifts in neutrophil function, we collected blood samples and measured NOC at three different physiological states within the torpor-arousal cycle: (i) early torpor, (ii) late torpor, and (iii) during interbout arousal upon arousals. With the intention of testing for a possible effect of temperatures on neutrophil function, we additionally reversed the respective NOC assay temperature (low torpid vs high euthermic) for each hibernating state. As we expected a substantial reduction of WBCs in torpor and a vast increase upon arousals, we additionally predicted that NOC would be significantly diminished at both temperatures during torpor followed by a reversal to a certain degree during interbout arousal.

## Materials and Methods

### Experimental Animals

Twenty-five garden dormice (11 males, 14 females), 1.5 to 2.5 years old, issued from a breeding colony kept at the Research Institute of Wildlife Ecology (Vienna, Austria), were included in the experiment. Garden dormice are small fat storing and omnivorous hibernators endemic to Europe (Mrosovsky and Lang, 1980). They are known for their naturally long hibernation periods which can be induced by decreasing their housing temperature and by limiting food availability. Garden dormice show very regular rhythms of torpor and arousal phases, another reason why this species is especially suited for hibernation research. In our main colony, animals are housed in large outdoor enclosures and exposed to natural fluctuations of T_*a*_ and photoperiod. During winter (October to March), dormice are naturally hibernating, and when T_*a*_ is low (<10°C), animals enter multi-days torpor bouts, i.e., hibernation, and do not feed or drink during several months (Giroud et al., 2014; Mahlert et al., 2018). For this particular experiment, animals were housed in individual cages (60 × 40 × 40 cm), each equipped with one nest, bedding and nesting material as well as branches with leaves, already during the pre-hibernation period. These cages were set up in a room under natural photoperiod, at a constant T_*a*_ of 20°C, with *ad libitum* access to food and water. For the actual winter hibernation period, garden dormice were transferred to individual standard laboratory cages (36 × 20 × 14 cm), each provided with a customized nest for proper hibernation kept at 4°C in ventilated cooling units (refrigerators; Liebherr GKv 5730) under constant darkness, without food and water, as previously described (Giroud et al., 2018; Logan et al., 2020).

### Ethics Statement

All procedures were approved by the institutional ethics committee and the Austrian national authority according to §26 of the Animal Experimental law, Tierversuchsgesetz 2012 – TVG 2012 (BMWF – 68.205/0137-WF/V/3b/2014).

### Protocol Overview

Before hibernation experiments, we implanted our study animals with small temperature transmitters, and core T_*b*_ was monitored via a telemetry system (read below for further details and specifications). Once animals were spontaneously entering prolonged (>24 h) torpor, hibernation was induced by housing the animals at 4°C without food and water. Hibernation was monitored during the next 3 months until animals were sacrificed at mid-winter (Dec 2014–Jan 2015) where torpor bout lengths were maximal (12.1 ± 1.4 [9.2 to 15.5 days CI]). Torpid animals were sacrificed by immediate decapitation. Euthermic animals were quickly removed from the cooling unit and were then incrementally exposed (flow rate regulator) to carbon dioxide (CO_2_, 100%) in a standard makrolon cage (type II long) with a modified lid (Corbach, 2006). Exposure lasted for 20 s at 3 L/min, then 40 s at 6 L/min until loss of consciousness followed by decapitation.

Torpor was defined when T_*b*_ of the animals decreased below 18°C, which was also the threshold to consider individuals above this value as euthermic. Torpid animals were sacrificed either in early torpor (1.5 ± 0.3 days torpid), in late-torpor (9.5 ± 0.2 days torpid) or during interbout arousal when they were euthermic for 3.4 ± 1.2 h. Fresh blood was collected in lithium heparinized tubes (LiHep Micro-tube, 1.3 ml, Sarstedt, Germany) immediately after decapitation via the trunk of the animals for immediate measurements of NOC and subsequent assessments of hematological parameters.

### Measurement of Core Body Temperature

Before implantation T_*b*_ transmitters (model: TA-10TA-F10, 1.1cc, 1.6 g, accuracy: 0.15°C; Data Sciences International, St Paul, MN, United States^1^) were calibrated for temperatures between 0 and 40°C in a temperature-controlled water bath. Transmitters were surgically implanted under anesthesia induced by subcutaneous injection of 50 mg kg^–1^ ketamine (Ketamidor 10%, Richter Pharma, Wels, Austria) and 5 mg kg^–1^ xylazine (Rompun 2%, Bayer, Leverkusen, Germany), as routinely performed in garden dormice (Logan and Storey, 2016; Giroud et al., 2018; Mahlert et al., 2018). Anesthesia was maintained with 1.5% isoflurane via an oxygen stream through a facemask. For postoperative analgesia 5 mg kg^–1^ ketoprofen (Romefen 10% Merial S.A.S., Toulouse, France) was administered subcutaneously. The operation field was prepared according to standard surgical procedures and covered by sterile surgical drapes. Animals were placed in dorsal recumbency, and the abdominal cavity was opened through a 1 cm incision in the *linea alba* to introduce the temperature transmitter into the abdomen. Peritoneum and abdominal muscles were sutured using synthetic absorbable surgical suture material USP 3/0 (Surgicryl PGA, SMI AG, Hünningen, Belgium) using a single-button suture technique. For the additional intra-cutaneous skin suture, we used synthetic absorbable surgical suture material USP 4/0 (Surgicryl PGA, SMI AG, Hünningen, Belgium). All standard vital parameters [respiration rate, peripheral hemoglobin oxygen saturation as measured by pulse oximetry (SpO_2_), and heart rate] were monitored during the entire procedure. After surgery, all animals recovered for 10 days before starting temperature recordings. For real-time temperature readings, a receiver board (RPC-1; Data Sciences International, St Paul, MN, United States; see text footnote 1) was positioned under every individual cage to collect the radio frequency signals from transmitters. T_*b*_ was recorded for 10 sec every 5 min. The Dataquest software package (LabPro Data Sciences) was used to download and visualize the data for first inspections.

### White Blood Cell Differential Count

An aliquot of lithium-heparinized full blood was sent to the *INVITRO* laboratory (*Invitro* – Laboratory for Veterinary Diagnostics and Hygiene GmbH, Vienna, Austria) for WBC-differential counts. WBC differential counts were done manually from Diff-quick stained blood smears by a clinical pathologist from the *INVITRO* laboratory. Total leukocytes were analyzed from all individuals (early torpor, *n* = 8; late torpor, *n* = 9; interbout arousal, *n* = 8). Due to hemolysis of some blood samples, neutrophils and lymphocytes (early torpor, *n* = 7) as well as monocytes (early torpor, *n* = 4; late torpor, *n* = 5; interbout arousal, *n* = 6) and eosinophils (early and late torpor, interbout arousal, *n* = 5) could not be differentiated and the sample size was reduced in these parameters.

### Neutrophil Oxidative Burst Capacity (NOC)

To measure individual baseline levels of ROS, i.e., unstimulated blood chemiluminescence levels as well as the oxidative burst capacity of neutrophils, we followed previously published protocols (Shelton-Rayner et al., 2012; Huber et al., 2019) which have been used for small rodents (Gelling et al., 2009, 2010). Briefly, 10 μl of lithium-heparinized whole blood was transferred into four silicon antireflective tubes (Lumivial, EG & G Berthold, Germany). We added 90 μl of 10^–4^ mol l^–1^ luminol (5-amino-2,3-dihydro-phthalazine-1,4-dione; VWR International, Stockholm, Sweden) dissolved in dimethyl sulfoxide (DMSO; Sigma-Aldrich (now Merck), Darmstadt, Germany) and diluted with phosphate-buffered saline (PBS, pH 7.4) in each tube. Luminol is excited by phagocyte-generated ROS, resulting in chemiluminescence (Merényi et al., 1990). Subsequently, in two tubes acting as the unstimulated individual control sample, 10 μl of PBS was added, and the tubes were swiveled gently for mixing. To measure full blood chemiluminescence produced in response to a chemical challenge 10 μl of 10^–5^ mol l^–1^ phorbol 12-myristate 13-acetate (PMA; Sigma-Aldrich (now Merck), Darmstadt, Germany) was added into the other two tubes instead of 10 μl PBS. PMA is a well-characterized activator of protein kinase C and neutrophil oxidative burst inducer (Karlsson et al., 2000). All measurements were carried out directly after the blood sample was collected in a closed experimental room, ensuring stable conditions at 20°C. Immediately after blood collection and preparation of the samples (<1 min), tubes were incubated for 2 min at either 6 or 35°C (one baseline and one challenge sample for each temperature regime) in a lightproof water bath before starting the measurements. Blood chemiluminescence was assessed using a portable high sensitivity chemiluminometer (Junior LB 9509, EG & G Berthold, Germany). Measurements for each tube were made at the start (i.e., 0 min) and every 5 min for a total of 30 s over 30 min, resulting in 7 measure time points expressed in relative light units (RLU) per individual and temperature treatment. When not in the chemiluminometer, tubes were incubated at the respective temperature regime, i.e., at 6°C (“low”) or at 35°C (“high”).

In order to correct for background noise, we subtracted the values of the control sample from that of the challenged sample measured at the same time-point. The obtained values were used for the subsequent statistical analysis.

### Data Computations and Statistical Analysis

All statistical analyses were carried out in R 3.6.2 (R Core Team, 2019). To test for an effect of hibernating states on body temperature, the total number of WBCs, neutrophils, lymphocytes, monocytes and eosinophil granulocytes, we constructed separate linear models for each variable that only contained hibernation state as the solitary explanatory variable (function lm).

To analyse the NOC an integral of the area under the curve was calculated based on the seven measured chemiluminescence-assay time points for each individual at each temperature treatment, and was used for subsequent analyses (function “rollmean”; package “zoo”; Zeileis and Grothendieck, 2005). To test for a potential effect of the different hibernating states (early and late torpor as well as interbout arousal), the two different temperature treatments (6°C vs 35°C), and the interaction of these two variables on the NOC a linear mixed-effects model was constructed (function “lme”; package “nlme” Pinheiro et al., 2017). Within this model, we additionally included the number of neutrophils for the respective individual sample in order to correct for a potential mass effect on ROS production as well as animal ID as a random effect. For all models normal distribution of residuals was inspected using histograms and quantile–quantile plots. In case of deviations from normality, data were boxcox-transformed, as the respective retested models showed no violation of model assumptions thereafter.

All constructed models were analyzed statistically with ANOVAs with type II sum of squares (function Anova, package “car” Fox and Weisberg, 2018). *Post hoc* comparisons between hibernating states were carried out with Tukey-like tests (function “glht” package “multcomp” Hothorn et al., 2008). All reported values are expressed as means ± SD.

## Results

### Body Temperatures of Hibernating Garden Dormice

**FIGURE 1:**
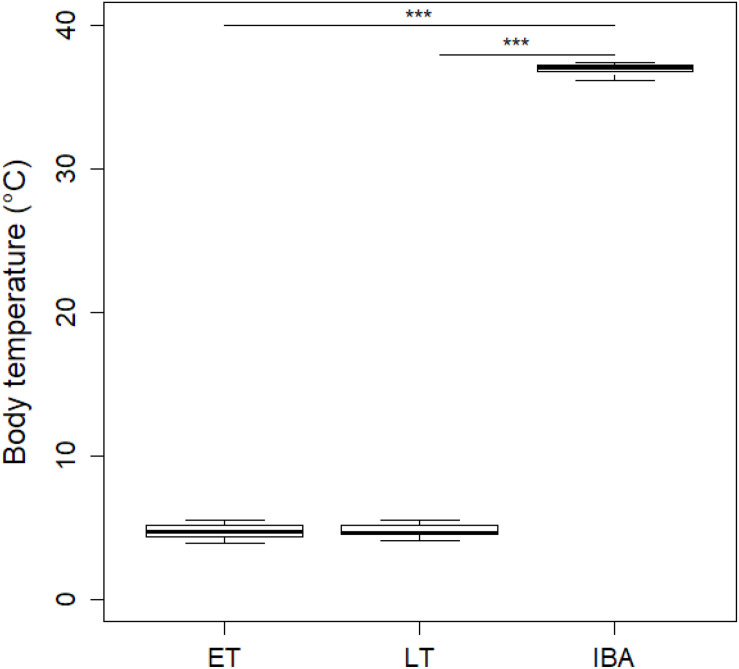
Boxplot of mean core body temperatures (T_*b*_) of hibernating garden dormice. Core T_*b*_ of animals in early torpor (“ET,” *n* = 8) was 4.73 ± 0.54°C which did not significantly differ from that of animals in late torpor (“LT”; 4.78 ± 0.51°C; *n* = 9; estimate = 0.04 ± 0.24, *z* = 0.16, *p* > 0.98). In contrast, euthermic animals during interbout arousals (“IBA,” *n* = 8) had significant higher T_*b*_ values of 36.96 ± 0.39°C compared to individuals in ET (estimate = 32.22 ± 0.24, *z* = 132.24, *p* ≤ 0.001) or LT (estimate = 32.18 ± 0.24, *z* = 135.91, *p* ≤ 0.001). Asterisks indicate significant differences (*p* ≤ 0.001) between physiological hibernating states.

During early torpor, dormice had a mean T_*b*_ of 4.73 ± 0.54°C which did not significantly differ from that of animals in late torpor with a T_*b*_ of 4.78 ± 0.51°C; Figure 1). As expected, interbout arousal euthermic animals showed a significant T_*b*_ increase of 36.96 ± 0.39°C compared to torpid individuals in early or late torpor (Figure 1).

### Levels of White Blood Cells (WBC) of Hibernating Garden Dormice

We found significant shifts in total WBCs and WBC differential counts concerning neutrophils and lymphocytes. Range (minimum, maximum), means and standard deviations of circulating WBCs during the phases of early and late torpor as well as interbout arousal, are shown in the Table 1.

**TABLE 1:** Range (minimum and maximum), means and standard deviations of total and differential white blood cell counts as well as the neutrophil oxidative burst capacity (NOC, measured at 6 and 35°C) at three different physiological states within the torpor-arousal cycle of hibernating garden dormice.

**State**	**ET (*n* = 8)**				**LT (*n* = 9)**				**IBA (*n* = 8)**			
	**min**	**max**	**mean**	**± SD**	**min**	**max**	**mean**	**± SD**	**min**	**max**	**mean**	**± SD**
Leukocytes G/L	0.90	4.08	1.88	1.13	1.05	5.35	2.21	1.41	2.91	7.83	5.62	1.53
Neutrophils G/L*	0.03	0.60	0.32	0.23	0.15	1.93	0.84	0.61	1.28	5.25	2.87	1.32
Lymphocytes G/L*	0.38	3.28	1.49	1.15	0.49	3.32	1.24	0.95	1.38	4.52	2.58	0.99
Monocytes G/L**	0.02	0.04	0.03	0.01	0.02	0.11	0.06	0.04	0.05	0.54	0.17	0.19
Eosinophils G/L^∗∗∗^	0.01	0.57	0.19	0.24	0.02	0.45	0.16	0.17	0.03	0.16	0.08	0.05
AUC_NOC_6°C	0.00	260.00	82.81	81.25	32.50	790.00	128.33	242.23	30.00	177.50	77.19	52.36
AUC_NOC_35°C	10.00	272.50	100.94	88.30	75.00	3550.00	608.33	1121.04	1057.50	6642.50	3768.44	2041.11

Blood samples were collected during the states of early torpor (“ET”; 1–2 days torpid; T_*b*_ mean ± SD = 4.73 ± 0.54°C), late-torpor (“LT”; 9–10 days torpid; T_*b*_ = 4.78 ± 0.51°C), and interbout arousal (“IBA”; 3.4 ± 1.2 h after arousal; T_*b*_ = 36.96 ± 0.39°C). “AUC_NOC_6°C” and “AUC_NOC_35°C” represent the areas under the curve response of neutrophil reactive oxygen species production measured every 5 min over 30 min at either 6 or 35°C. G/L = 10^9^/l. *n = 7 in ET. **n = 4 in ET, 5 in LT, 6 in IBA. ***n = 5 in ET, LT, and IBA.

**FIGURE 2:**
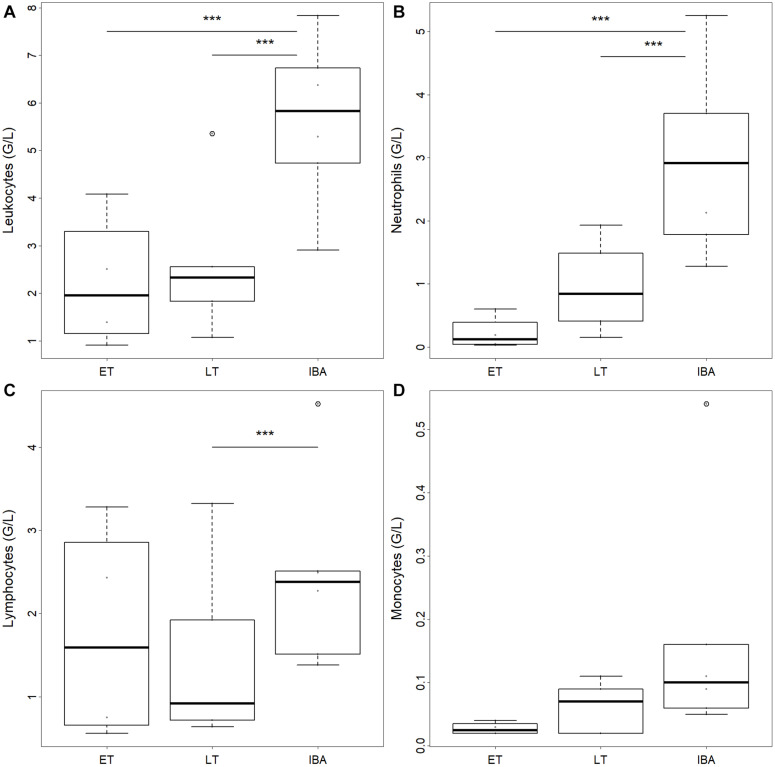
Box plots of circulating **(A)** total leukocyte, **(B)** neutrophil, **(C)** lymphocyte, and **(D)** monocyte numbers in hibernating garden dormice under different physiological states within the torpor-arousal cycle. Hibernating states correspond to early torpor (“ET,” *n* = 8), late-torpor (“LT,” *n* = 9), and interbout arousal (“IBA,” *n* = 8). Asterisks indicate significant differences between states (*Post hoc* Tukey-like test: ***p < 0.001). When not indicated, effects were non-significant.

The number of total WBCs in average tripled during interbout arousal compared to torpid animals without difference between early and late torpor (Table 2 and Figure 2A). Neutrophils increased 3.6-fold on average during interbout arousal compared to animals in the early or late phase of torpor with no significant difference between early and late torpor (Table 2 and Figure 2B). Lymphocyte numbers were significantly lower only during late torpor compared to interbout arousal but were not different between interbout arousal and early torpor (Table 2 and Figure 2C). Also, no significant differences between hibernating states were found for monocyte (Table 2 and Figure 2D) or eosinophil counts (Table 1).

**TABLE 2:** Differences in total and differential white blood cell count between three different measured time points within the torpor-arousal cycle in hibernating garden dormice.

	**Estimate**	**Std. Error**	***t*-value**	***p*-value**
**Leukocytes**				
*F*_2_,_51_ = 18.642, *p* < 0.001				
LT–ET	0.329	0.664	0.495	0.771
IBA–ET	3.742	0.683	5.473	**<0.001**
IBA–LT	3.413	0.664	5.137	**<0.001**
**Neutrophils**				
*F*_2_,_24_ = 19.064, *p* < 0.001				
LT–ET	0.518	0.434	1.192	0.471
IBA–ET	2.551	0.446	5.722	**<0.001**
IBA–LT	2.034	0.419	4.858	**<0.001**
**Lymphocytes**				
*F*_2_,_24_ = 4.321, *p* = 0.026				
LT–ET	−0.043	0.290	−0.145	0.878
IBA–ET	0.727	0.305	2.377	0.127
IBA–LT	0.770	0.287	2.682	**0.036**
**Monocytes**				
*F*_2_,_15_ = 1.86, *p* = 0.198				
Eosinophils				
*F*_2_,_15_ = 0.545, *p* = 0.593				

*Lymphocyte numbers were boxcox transformed to meet the assumption of statistical normality. Differences are reported as partial effects accounting for the individual as a random effect. Blood samples were collected during the physiological states of early torpor (“ET,” *n* = 8), late-torpor (“LT,” *n* = 9), and interbout arousal (“IBA,” *n* = 8). It was not possible to differentiate neutrophils and lymphocytes due to hemolysis in one blood sample and the sample size for these parameters during early torpor are reduced by one (*n* = 7). Significant differences are displayed in bold letters.*

### Neutrophil Oxidative Burst Capacity (NOC) of Hibernating Garden Dormice

**FIGURE 3:**
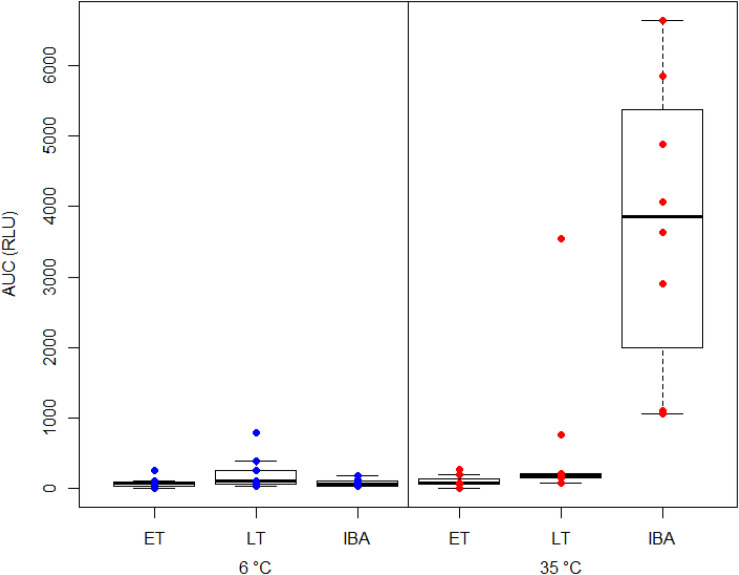
Box plot showing the area under the curve response (“AUC”) expressed in relative light units (“RLU”) of the neutrophil oxidative burst capacity (NOC) in hibernating garden dormice under different physiological states within the torpor-arousal cycle. Blood samples from dormice in early torpor (“ET,” *n* = 8), late-torpor (“LT,” *n* = 9), and interbout arousal (“IBA,” *n* = 8) were measured at both 6 and 35°C. NOC from IBA animals measured at 35°C is significantly higher compared to those from all other physiological states and measurement temperatures (*p* < 0.001). Please note the higher variability of NOC response from the blood of LT animals measured at 35°C.

**FIGURE 4:**
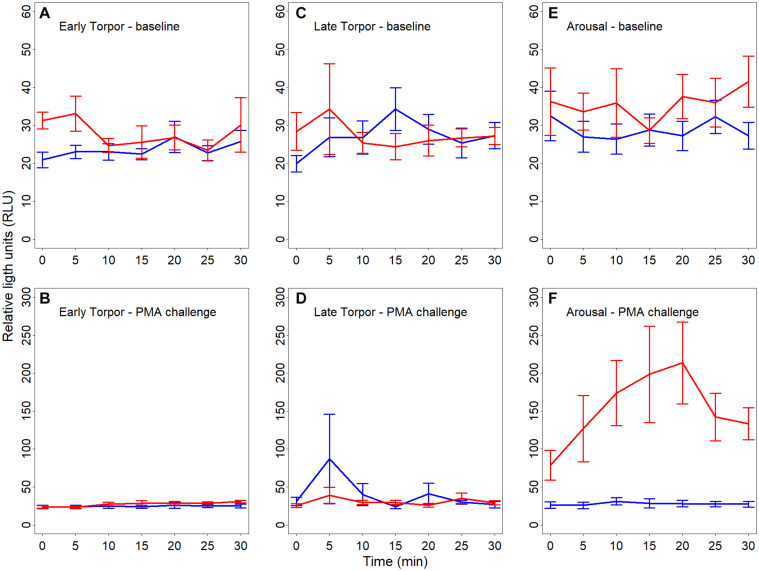
**(A–F)** Line plots showing baseline reactive oxygen species (ROS; **A**,**C**,**E**) as well as challenge ROS levels **(B,F,F)** in full blood of hibernating garden dormice measured for 30 s every 5 min over 30 min and expressed in relative light units (“RLU”). Chemiluminescence was measured in different physiological states within the torpor-arousal cycle, i.e., in early torpor (**A**,**B**; *n* = 8), late-torpor (**C**,**D**; *n* = 9), and interbout arousal (**E**,**F**; *n* = 8) at both 6°C (blue lines) and 35°C (red lines). There was no significant difference in baseline chemiluminescence between the different states (all *p* > 0.5) with a small increase in baseline ROS in IBA animals when measured at 35°C. Error bars represent the standard error.

We found a significant interaction between hibernating states and temperature treatments during the NOC assay (Table 3 and Figure 3). *Post hoc* tests revealed significantly higher NOC levels in interbout arousal dormice, measured at 35°C, compared to those from torpid animals measured at 6°C. NOC Levels of animals in interbout arousal measured with the reversed temperature, i.e., at 6°C, significantly dropped compared to the measured levels at 35°C, and then did not significantly differ from the ones of the torpid animals (Table 3 and Figure 3). Range (minimum, maximum), means and standard deviations for NOC values for the respective assay temperatures during in the phases of early and late torpor as well as interbout arousal are presented in Table 1. Interestingly, we observed no significant effects of the assay’s temperature on NOC of either early or late-torpor individuals, all having the same low amplitudes (Table 3 and Figure 3). The full NOC response curves over the 30-minute measure period including the baseline as well as the challenge sample for each assay temperature during early- and late torpor and during interbout arousal are presented in Figure 4A–F. Further, testing for a potential mass effect of neutrophils on NOC revealed that the NOC response was independent of the number of neutrophils (X ^2^_2_,_48_ = 0.45, *p* = 0.50; Table 2). However, we did observe a positive but non-significant correlation between the number of neutrophils and the NOC response in animals during interbout arousal (for visualization please see Supplementary Figure 1).

**TABLE 3:** Differences in neutrophil oxidative burst capacity (NOC) between three different time points, i.e., early torpor (“ET”), late-torpor (“LT”), and the interbout arousal (“IBA”), within the torpor-arousal cycle of hibernating garden dormice.

	**Estimate**	**Std. Error**	***z*-value**	***p*-value**
**Hibernation state: Temperature**				
X ^2^_2_,_48_ = 38.96, *p* < 0.001				
IBA 35°C–ET 6°C	7.14	1.30	5.48	**<0.001**
IBA 35°C–LT 6°C	5.41	1.14	4.75	**<0.001**
IBA 35°C–IBA 6°C	7.21	0.82	8.81	**<0.001**
IBA 35°C–ET 35°C	6.33	1.30	4.85	**<0.001**
IBA 35°C–LT 35°C	4.33	1.14	3.80	**0.001**
All other comparisons *p* > 0.2				
**Neutrophils**				
X ^2^_2_,_48_ = 0.45, *p* = 0.50				

*NOC values were boxcox transformed to meet the statistical assumption of normality. Differences are reported as partial effects including the number of neutrophils for the respective individual to correct for a potential mass effect on ROS production as well as animals ID as random effect. NOC was measured at two different assay temperatures close to the core body temperature of either 35°C (euthermic) and 6°C (torpid). The number of neutrophils for the respective individual sample was included in the analysis in order to correct for a potential mass effect on NOC. Significant differences are displayed in bold letters.*

## Discussion

In this study, we assessed potential differences in the number and composition of WBC, as well as in the function, i.e., capacity to produce ROS, of neutrophils in garden dormice hibernating under controlled but natural conditions. We collected blood samples at three defined time-points within the torpor-arousal cycle, i.e., either early or late during the ultra-metabolic downstate of torpor, and after natural periodic arousal in interbout arousal. To test for a possible effect on NOC, beyond temperatures, we also reversed the respective NOC measurement temperature for each hibernating state. Besides a substantial drop in circulating WBCs accompanied by a shift in WBC composition during torpor reversible in interbout arousal, our results revealed a significant decrease in neutrophil function, i.e., NOC during torpor which was restored to a substantial degree in interbout arousal and the concomitant rise in T_*b*_ upon arousal.

### White Blood Cell Number and Composition Change Within the Torpor-Arousal Cycle

The observed extreme and transient leukopenia in our study animals during early and late torpor, including granulocytes (neutrophils, eosinophils), lymphocytes and monocytes, with a significant increase in WBCs to a certain level upon arousal (Figure 2A), confirms previous findings on WBC dynamics during hibernation from all hibernating species studied in this aspect to date (Bouma et al., 2010a,b, 2011, 2012, 2013b; Sahdo et al., 2013; Kuznetsova et al., 2016; Kizhina et al., 2018; Uzenbaeva et al., 2019). At this point, we would like to note that we did not measure summer euthermic animals in this study and, therefore, cannot draw any conclusions or comparisons on the seasonal aspect of blood cell counts or distribution.

Bouma et al. (2011) showed that lymphopenia, i.e., a marked drop of lymphocytes, during hibernation is driven by T_*b*_, via altered erythrocyte generated plasma sphingosine-1-phosphate (S1P) levels inducing the storage of lymphocytes in secondary lymphoid organs. In another study, it was demonstrated that the reversible neutropenia during torpor is primarily caused by margination neutrophils, i.e., the reversible attachment of blood cells to the endothelial wall of the blood vessels and that this phenomenon is not restricted to hibernating species (Bouma et al., 2013a; Sahdo et al., 2013).

In contrast to previous studies, the number of lymphocytes observed in our studied dormice during early torpor was similar or only moderately reduced compared to interbout arousal and only decreased to a significant level during late torpor (Figure 2C). Hence our results in regards to WBC composition in hibernating garden dormice do not fully align with previous studies. Bouma et al. (2010a) noted in their review that the majority of the remaining WBCs during the state of torpor are neutrophils (90%), whereas in our study lymphocytes are the predominant WBC type during early (71%) as well as during late torpor (52%), which is likely to be species-specific (see also Uzenbaeva et al., 2019). The lymphocyte numbers in our study are not significantly different between interbout arousal and early torpor, i.e., 1–2 days after interbout arousal, but continue to decrease further along the torpor bout. One possible explanation may be that S1P levels in early torpor are only minimally lower compared to levels during interbout arousal but decrease over the course of torpor with the consequence of a further reduction of lymphocytes and the observed significant differences in lymphocyte abundance between late torpor and interbout arousal. A higher proportion of lymphocytes to phagocytes, i.e., neutrophils and monocytes, was also reported in edible dormouse (*Glis glis*) shortly after emergence from hibernation in spring (Havenstein and Fietz, 2013). Therefore, the higher proportion of lymphocytes during torpor in garden dormice may also have an immune protective role as shown for torpid 13-lined ground squirrels (*Ictidomys tridecemlineatus*), where the ability to mount a humoral immune response to a T-lymphocyte dependent antigen (NP-ovalbumin) remained optimal during hibernation (Bouma et al., 2013b). Moreover, the immunization of hibernating ground squirrels with NP-ovalbumin induced the production of antibodies and caused a disturbance of the hibernating pattern, i.e., an atypical (emergency) rewarming from torpor, along with increased mortality in this small hibernator (Bouma et al., 2013b). The results of the latter study demonstrate functional and responsive lymphocytes during torpor and the hibernation period and may partly explain the adaptive significance of maintaining a high proportion of lymphocytes during the hypo-metabolic downstate of torpor. Here we would like to note that the use of CO_2_ for euthanasia can impact blood cell distribution with increasing number of lymphocytes with increasing CO_2_ levels in the circulation (Pecaut et al., 2000). This may have influenced blood cell distribution in the interbout arousal group to a certain degree.

From an immunological perspective, our findings extend earlier work reporting that organismal immune cell responses are inhibited to varying degrees during torpor and restored close to summer euthermic levels during interbout arousal (Shivatcheva et al., 1988; Novoselova et al., 2000; Bouma et al., 2010b). In golden-mantled ground squirrels (*Spermophilus lateralis*) the acute phase response to bacterial lipopolysaccharide (LPS) was ultimately arrested during torpor but fully restored upon interbout arousal as animals developed a fever only during the subsequent interbout arousal which was additionally prolonged up to six times compared to regular interbout arousal durations in this species (Prendergast et al., 2002). In the latter study, Prendergast et al. (2002) also tested the neuronal signaling pathways mediating febrile responses with intra-cerebro-ventricular infusions of prostaglandin-E2 and demonstrated that the underlying neuronal circuit is functional during torpor and thereby narrowed the absent response to LPS during torpor down to cellular immune processes including LPS binding and cytokine production.

### Neutrophil Oxidative Burst Capacity (NOC) Reveals Restoration of Rapid Innate Immune Defense During Arousals

Studies on the NOC in non-hibernating species in mammals as well as in birds using the very same protocol as applied in present study observed a significant correlation between the number of neutrophils and NOC (Huber et al., 2019, 2020). The missing correlation in the current manuscript is likely explained, beyond the relatively small sample sizes, by the fact that a high number of neutrophils is only present in the IBA group (Supplementary Figure 1). Our results of NOC show a low to negligible ROS production in torpid animals when measured at a low temperature (close to torpid T_*b*_) with no significant difference between early and late torpor. Interestingly, and following our prediction, NOC of animals in interbout arousal was significantly increased up to 30-fold compared to the one measured in torpid individuals (Figure 3). An early study comparing the effect of temperature on NOC using whole-blood chemiluminescence in two non-hibernating species, the ectothermic rainbow trout (*Salmo gairdneri*) and humans (endotherms), revealed that the chemiluminescence responses were approximately equivalent for the two species measured at their physiological T_*b*_ associated with their respective preferred T_*a*_ (Sohnle and Chusid, 1983). The highest magnitude in the oxidative burst of human neutrophils was attained from 23 to 37°C, and between 4 and 15°C in rainbow trout neutrophils. When assay temperatures were reversed, i.e., chemiluminescence of human cells was measured at 4°C and trout cells at 37°C, the responses were negligible in both species (Sohnle and Chusid, 1983). Hence, the NOC appears to be temperature-sensitive but well adapted to the respective T_*b*_ and environmental T_*a*_ of each species. However, in mammals, the NOC response to soluble chemicals (e.g., PMA) as well as to phagocytic stimuli in general, has been shown to develop more rapidly and to a greater extent at higher T_*b*_ (Wenisch et al., 1996). Here we would like to remark, that also increased circulating CO_2_ levels can negatively influence the NOC response as reported in rainbow trout (*Oncorhynchus mykiss*) tissues (Kaya et al., 2013). Hence the NOC response in interbout arousal animals in our study may have been higher as measured in our study until the animals were exposed to increased CO_2_ levels (for 60 s) in the course of the sacrification procedure.

Also, in our system, the question regarding the drivers and the downstream mechanisms leading to the highly diminished NOC during the hypometabolic downstate of torpor arises. Reversing of the NOC measurement temperatures from animals in interbout arousal resulted in a significant reduction in NOC to an almost non-detectable level indicating a temperature dependence of the neutrophil oxidative burst response during euthermia (Figure 3). Conversely, the reversal of the measurement temperature from torpid animals, i.e., the rewarming of blood and measuring NOC close to euthermic temperatures, did not affect an increase in NOC and ROS production over the 30-min measurement period. The insignificant change of NOC in rewarmed blood from torpid animals let us speculate that the inhibition of NOC during torpor may be temperature-compensated. However, we would like to note that blood samples from animals were taken when animals had been euthermic for more than 3 h and the reversal (i.e., warming and cooling) of the assay temperatures occurred relatively fast and over only 32 min. This time difference (32 min vs 3 h) in cooling and rewarming rates may have influenced cell homeostasis and their ability to produce and release ROS differently, although the peak increase in metabolic rate, hence systemic oxidative burst, upon natural arousal from torpor occurs within the first 40 min of rewarming. In this context, the generation of microbicidal oxidants by neutrophils results from the assembly of the multiprotein enzyme complex nicotinamide-adenine-dinucleotide-phosphate- (NADPH) oxidase (NOX), which catalyzes the formation of superoxide anion, the primary molecule for various ROS (Babior, 1999; Quinn and Gauss, 2004). NOC by NOX is an NADPH-dependent process derived from the pentose phosphate shunt dependent on both glucose/glycogen and adenosine triphosphate for its formation (Ying, 2008). Therefore, one possible explanation for the diminished NOC in the torpid state could be the low substrate availability and the concomitant low levels of NADPH as a result of the extreme metabolic suppression during torpor.

The chemical stimulus PMA is a broad-spectrum protein kinase C (PKC) activator that bypasses receptor-mediated control of free radical production (Karlsson et al., 2000). PKC phosphorylates the NOX subunit p47^*PHOX*^ and thereby contributes to the assembly of the NOX and the subsequent production of ROS (Dang et al., 2006; Rastogi et al., 2017). Although we cannot conclude on the exact cause of the reduction or suppression in NOC during torpor, the regulation could specifically act via an insensitivity of PKC to PMA, altered subsequent activation of the NOX enzyme complex, or a combination of both (Babior, 1999). Also, an alternative explanation for the low NOC during torpor may be the inhibiting effect of hydrogen sulfide (H_2_S) on NOX and the linked ROS production (Muzaffar et al., 2008a,b; Al-Magableh et al., 2014; also see for review Giroud et al., 2020). Talaei et al. (2012) report endogenous production of H_2_S in hibernating Syrian hamsters (*Mesocricetus auratus*), with increased levels during early and late torpor and a normalization, i.e., low levels, during interbout arousal. Hence, the observed dynamic in NOC during different hibernating states may result from a similar dynamic of H_2_S levels along the torpor arousal cycle. Additionally, H_2_S is limiting the migration of neutrophils through tissues, suppresses the inflammatory response and is acting as an endogenous regulator of the innate as well as the adaptive immune system (Faller et al., 2018).

### Further Considerations on the Neutrophil Oxidative Burst Response During Hibernation

Luminol, the light enhancer used in this study, can diffuse across biological membranes and therefore allows the detection of both extra and intracellular ROS production (Briheim et al., 1984). However, several authors highlight that Luminol reflects intracellular ROS kinetic production rather than extracellular ROS detection (Müller-Peddinghaus, 1984; Caldefie-Chézet et al., 2002; Freitas et al., 2009). Furthermore, luminol chemiluminescence in full blood is a myeloperoxidase (MPO) requiring reaction and its presence and functionality depict another factor that could potentially interfere with the production and subsequent detection of ROS by neutrophils during the torpor-arousal cycle. In this context, it has also been shown that H_2_S inhibits MPO activity and the production of ROS by neutrophils and thereby contributes to an anti-neutrophil (anti-inflammatory) state. To disentangle the involved pathways and down-stream mechanisms, further studies comprising the use of additional chemiluminescence amplifiers such as iso-luminol, which is not able to cross cell membranes, or lucigenin, which is highly specific to the activity of the NOX complex and the production of extracellular ROS, are warranted (Dahlgren and Karlsson, 1999; Dahlgren et al., 2020). Furthermore, we recommend including the measurement of intra- and extracellular MPO concentrations using flow cytometry (Peake et al., 2004) as well as H_2_S levels as a promising candidate and underlying molecule regulating NOC along the torpor arousal cycle (Talaei et al., 2012; Dilek et al., 2020). Additionally, we suggest to include the measurement of PKC substrates such as p47^*PHOX*^ and their activity into future experiments and investigate the underlying mechanisms more in depth.

## Conclusion

To conclude, this study confirms changes in numbers, composition, and function of WBC during the alternating periods of extreme metabolic downregulation known as torpor and short euthermic periods of rapid and drastic increases in metabolic rate, called periodic arousals. Our study particularly reveals suppression of neutrophil function in terms of their capacity to produce oxygen radicals, a key-player of the innate immune system, which is reversed upon arousals when hibernators reactivate primary physiological functions, including the dormant immune system. Since WBCs and neutrophil granulocytes play a central role in the pathophysiology of tissue damage and organ injury, this dynamic may be obligatory to minimize inflammation and possibly organ injury during these extreme physiological shifts over the course of the torpor-arousal cycle and thereby to survive the hibernation period. Further studies are necessary to determine the underlying mechanisms and translate them into biomedical applications and therapies. Our findings highlight small hibernating species and natural hibernation in general as an excellent model to explore the adaptive value of immunological shifts during extreme hypometabolic and hypothermic states in the context of ischemia-reperfusion injury, organ preservation, and therapeutic hypothermia.

## Data Availability Statement

The raw data supporting the conclusions of this article will be made available by the authors, without undue reservation, to any qualified researcher upon request.

## Ethics Statement

The animal study was reviewed and approved by the Institutional Ethics Committee and the Austrian National Authority.

## Author Contributions

NH and SG conceived and designed the study. HG and GS performed the temperature logger implantations. NH performed all chemiluminescence assays and drafted the manuscript. SG and SV analyzed the data with input from NH. SG edited and critically revised the manuscript. All co-authors commented on the manuscript. All authors contributed to the article and approved the submitted version.

## Conflict of Interest

The authors declare that the research was conducted in the absence of any commercial or financial relationships that could be construed as a potential conflict of interest.

## Footnotes

1 https://www.datasci.com
