# Supplementary material for: Dynamic Function and Composition Shift in Circulating Innate Immune Cells in Hibernating Garden Dormice
Source: Front Physiol. 2021 Mar 4;12:620614. doi: 10.3389/fphys.2021.620614 (PMC7970003; doi:10.3389/fphys.2021.620614)
Supplement: Supplementary file 1 [file Data_Sheet_1.PDF]

Supplementary Material

Dynamic function and composition shifts in circulating innate immune cells in hibernating garden dormice

Nikolaus Huber[1,2*], Sebastian Vetter[3], Gabrielle Stalder[1], Hanno Gerritsmann[1], Sylvain Giroud[1,*]

[1]Research Institute of Wildlife Ecology, Department of Interdisciplinary Life Sciences, University of Veterinary Medicine, Vienna, Savoyenstraße 1, 1160 Vienna, Austria

[2]Unit of Veterinary Public Health and Epidemiology, Institute of Food Safety, Food Technology and Veterinary Public Health Department for Farm Animals and Veterinary Public Health, University of Veterinary Medicine Vienna, Austria

[3]Institute of Animal Welfare Science, Department for Farm Animals and Veterinary Public Health, University of Veterinary Medicine Vienna, Austria

***Correspondence:**
Corresponding authors NH and SG
emails: nikolaus.huber@vetmeduni.ac.at; sylvain.giroud@vetmeduni.ac.at
Phone: +43-1-25077-7270; Fax: +43-1-25077-94-7270

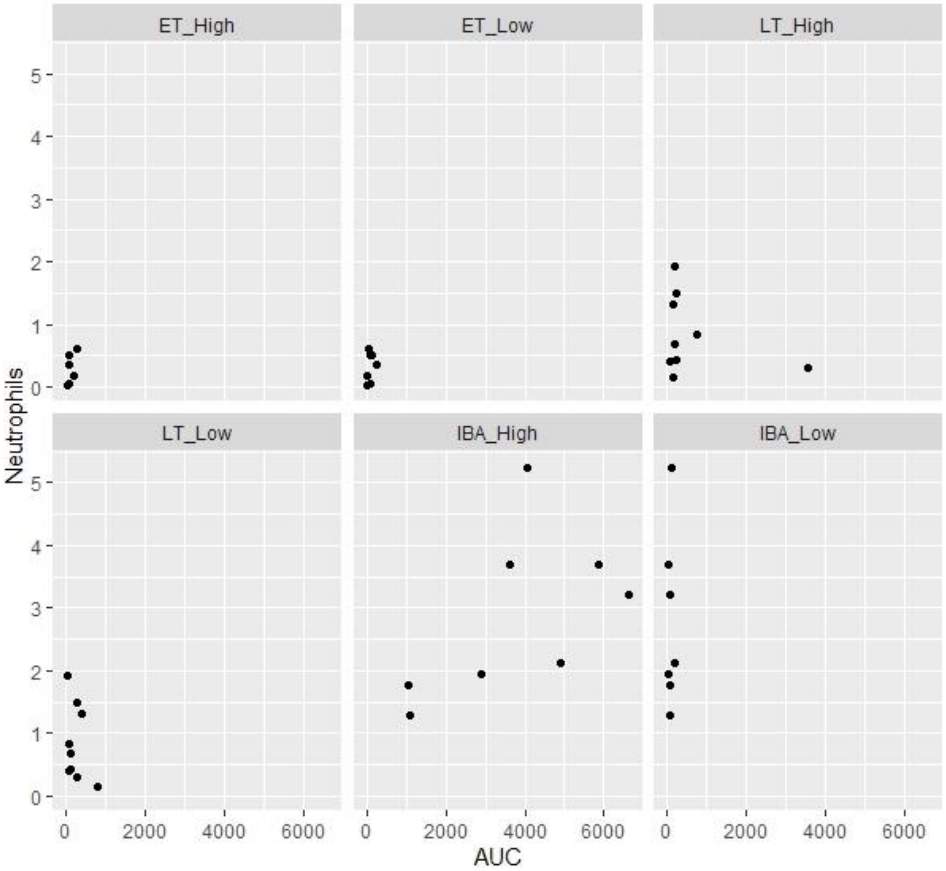

Supplementary Figure 1. Dot-plot showing the relation between the number of neutrophil granulocytes and the neutrophil oxidative burst response calculated as area under the curve (AUC) during early-torpor ('ET'; 1-2 days torpid), late-torpor ('LT'; 9-10 days torpid) and the interbout arousal ('IBA'; 3.4 ± 1.2h after arousal), within the torpor-arousal cycle of hibernating garden dormice. NOC was measured at two different assay temperatures close to the core body temperature of either 35°C (_High) or 6°C (_Low) for the respective hibernation state.
